# App- and Wearable-Based Remote Monitoring for Patients With Myasthenia Gravis and Its Specialists: Feasibility and Usability Study

**DOI:** 10.2196/58266

**Published:** 2025-03-03

**Authors:** Maike Stein, Regina Stegherr, Pushpa Narayanaswami, David Legg, Meret Herdick, Andreas Meisel, Lea Gerischer, Sophie Lehnerer

**Affiliations:** 1 Department of Neurology with Experimental Neurology, Charité – Universitätsmedizin Berlin, corporate member of Freie Universität Berlin and Humboldt-Universität zu Berlin, Charitéplatz 1, Berlin, 10117, Germany, 49 30450539778; 2 Digital Health Center, Berlin Institute of Health at Charité, Charité Universitätsmedizin Berlin, Berlin, Germany; 3 Department of Neurology, Beth Israel Deaconess Medical Center/Harvard Medical School, Boston, MA, United States; 4 Neuroscience Clinical Research Center, Department of Neurology with Experimental Neurology, Charité – Universitätsmedizin Berlin, corporate member of Freie Universität Berlin and Humboldt-Universität zu Berlin, Berlin, Germany; 5 Institute of Biometry and Clinical Epidemiology, Charité – Universitätsmedizin Berlin, corporate member of Freie Universität Berlin and Humboldt-Universität zu Berlin, Berlin, Germany; 6 Center for Stroke Research Berlin, Charité Universitätsmedizin Berlin, Berlin, Germany

**Keywords:** myasthenia gravis, myasthenia, remote monitoring, PROMs, digital platform, wearables, telemedicine, spirometry, app, usability, feasibility, autoimmune disorder, web-based portal, activity tracker, communication, wearable data, digital tool, mobile phone

## Abstract

**Background:**

Myasthenia gravis (MG) is rare, chronic autoimmune disorder of the neuromuscular junction that requires specialized care and often lifelong treatment, facing challenges due to its rarity and the limited availability of specialists. Telemedical solutions in specialized centers hold considerable promise in bridging this gap by increasing access to this care to a broader patient population in a timely manner. However, there is no research regarding interventional remote care solutions in the field of MG to date.

**Objective:**

This study aimed to assess the feasibility and usability among patients with MG and specialists of a telemedicine platform, tailored to patients with MG and designed to facilitate remote monitoring, treated in a specialized center.

**Methods:**

The telemedicine platform consisted of an app for patients and a web-based portal for physicians. Over a period of 3 months, 30 patients continuously monitored their vital parameters through external devices, including a digital spirometer and a wearable (activity tracker). Furthermore, patients completed 7 different patient-reported outcome measures (PROMs) through the app at predefined intervals. Specialists could review this monitoring data and adjust therapy as necessary. In addition, communication between patients and physicians was facilitated through a chat module. Feasibility was evaluated by total adherence rates for completing PROMs within the app, alongside the collection of spirometry and wearable data. Furthermore, user satisfaction was assessed among both patients with MG and physicians at the end of study.

**Results:**

Total adherence rates ranged from 74.3% (1830/2464) to 97.9% (327/334) across all data types, with the highest adherence observed for PROMs (1139/1179, 96.6%), followed by spirometry (293/334, 87.7%) and wearables (1830/2261, 80.9%). Notably, patients wore the wearable longer than required by protocol and conducted a higher number of spirometry measurements during the study than required per protocol (median 20 h/d [IQR 15-24] vs 14 h/d and median 49 [IQR 15-59] measurements vs 11 measurements, respectively). Technical issues and discomfort with wearables were factors affecting lower adherence in some patients. The System Usability Scale yielded a median score of 85 indicating “excellent usability.” In addition, results from a more detailed user evaluation questionnaire showed high levels of user satisfaction among both patients and health care professionals across diverse categories, including their experience of the care program, communication, and evaluation of the program.

**Conclusions:**

Remote monitoring of patients with MG through the telemedical platform demonstrated good feasibility and acceptability, as evidenced by above-average adherence rates and user satisfaction for both patients and physicians. The majority of patients wanted to continue using the app. These findings highlight the potential for user-friendly digital tools to enhance care for patients with MG, although addressing technical challenges and ensuring patient comfort with wearables are essential for optimal implementation. Further research involving larger cohorts and longer study duration is warranted to validate these findings.

## Introduction

Myasthenia gravis (MG) is a rare autoimmune disease characterized by specific autoantibodies targeting the postsynaptic membrane of the neuromuscular junction, resulting in fluctuating fatigability and weakness of ocular, bulbar, and skeletal muscles, with the potential for life-threatening crises [1]. MG has a bimodal incidence pattern, with younger women and older men being most commonly affected [2]. Given its chronic nature, the majority of patients with MG require long-term and often lifelong, highly specialized care and therapy [3]. Furthermore, studies have shown that patients with MG experience a significantly reduced quality of life [4] and have a high demand for individualized counseling on various topics [5] and supportive interactions. In addition, characteristic symptom fluctuations are not captured effectively by conventional clinical assessments that rely on infrequent in-person assessments that are further challenged due to documentation overload for physicians, limited consultation time, and geographical barriers that limit access to specialists [6] . The characteristic symptom fluctuations in MG, but also the long intervals for therapies to effectively influence the disease including potential side effects, give rise to questions and challenges for timely therapy adjustments and individual counseling. However, in practice, these adjustments often do not occur in a timely manner or are conducted in ways that pose data privacy problems, are resource intensive and lack standardization. These challenges and the growing demand for personalized and on-demand available health care have spurred the development of telemedical and wearable devices for remote patient monitoring [7]. Wearable devices such as activity trackers facilitate continuous monitoring of various health parameters and have shown promise in reducing hospitalization costs, improving personalized health management [8-10] and health‐related quality of life [11]. Those digital health interventions hold potential for bridging the care gap by offering individualized support based on continuous health data derived from both subjective patient-reported outcome measures (PROMs) and objective wearable and sensor-based data. Although previous remote digital studies have shown the feasibility and usability of leveraging smartphones and wearable technology for assessing clinical changes in different diagnosis and naturalistic settings [12-14], there is to date no data available on telemedical interventions for patients with MG. Generally, successful integration of these technologies into health care relies on patients’ active involvement and acceptance, underscoring the importance of digital literacy [15,16]. In the context of remote monitoring technologies, adherence serves as a crucial measure of patient compliance with study protocols (eg, the completion rate of providing data inputs at specified frequencies outlined in the protocol) and is dependent on their willingness and capacity to adopt new behaviors and practices. We hypothesize that the integration of digital tools into clinical care presents a viable approach for remotely monitoring the condition and symptoms of patients with MG. The aim of this study was to evaluate the feasibility and user satisfaction levels among patients with MG and health care professionals with a telemedicine platform specifically tailored to monitor patients with MG and enabling patient-physician interaction.

## Methods

### Telemedical Platform for Patients with MG

**Figure 1.**
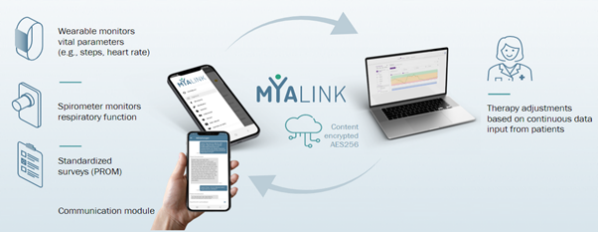
Schematic overview of telemedical platform and its features (MyaLink): transmission and simultaneous integration of sensor-based data into the app is implemented by bluetooth from coupled external devices (wearable or digital spirometer). In addition, the platform facilitates the collection of patient-reported outcome measures (PROMs) through the app and a communication module enables interaction between patients and physicians. Health data can be shared with specialists who can review monitoring parameters through the web-based system and make therapy adjustments as needed. The data is transmitted between patient and physicians with double encryption (AES256, Advanced Encryption Standard). PROM: patient-reported outcome measure.

The telemedical platform for patients with MG, “MyaLink” used in the study was developed at Charité Universitätsmedizin Berlin together with software partner Qurasoft GmbH (based on the SaniQ platform). The platform is a Conformité Européenne (CE)-marked class-I medical device (Medical Device Directive) and is GDPR (General Data Protection Regulation) and BlnDSG (Berlin Data Protection Act) compliant. The platform consists of an app for patients (iOS and Android) and a web-based portal for physicians facilitating to review patient data, manage and set up individual monitoring plans and medication plans. It offers a secure interactive platform for exchange of information and health data between physicians and patients and informed treatment decisions (Figure 1). The MyaLink app contains the following modules: (1) transmission and integration of sensor-based data from wearables including heart rate, oxygen saturation, and step count (device: Garmin Vivosmart 5); (2) assessment of the forced vital capacity from a digital spirometer (device: MIR Spirobank Smart One); (3) assessment of questionnaires and PROMs (see details under Section 3.5); and a (4) communication module for individual requests and patient-physician interaction. Furthermore, the app contains a (5) medication plan with reminder function and (6) a module including data storage of important documents (eg, clinical reports), which can also be shared with the physician through the web-based portal. The web-based system for physicians enables real time access to this data and allows clinicians to obtain an accurate overview of the patients’ condition and visualization of health data. If necessary, the patient can be contacted through the communication tool and adjustments to medication and the remote monitoring plans can be made.

### Subjects’ Eligibility

Inclusion and exclusion criteria have been previously presented in detail in our methods paper [17]. Inclusion criteria included patients with a diagnosis of MG for at least 6 months before study enrollment, aged 18 years and older, and classified according to the Myasthenia Gravis Foundation of America (MGFA) classification status I-IV [18]. Exclusion criteria included patients with somatic, psychiatric, or neurological disorders who were undergoing treatments or were not stabilized under drug therapy that could adversely affect cognitive function. Patients were recruited through the specialized outpatient myasthenia center at Charité Universitätsmedizin Berlin.

### Study Design

This analysis focused on data obtained from the telemedical intervention group from a randomized controlled trial (RCT) conducted at our specialized center at Charité Universitätsmedizin Berlin in Germany. The RCT included 45 patients with MG over a 12-week observation period between April and September 2023. Patients were randomized in a 2:1 ratio into the telemedical intervention group (n=30) or the control group (n=15), who received standard of care without telemedical intervention. Randomization was performed according to gender and clinical class stratified by MGFA score at baseline (using the randomizer tool [19]). The primary objective of the study was to investigate the feasibility of the remote monitoring platform in care of patients with MG. Feasibility was measured through adherence rates based on completion of PROMs in the app and data collection from external devices (including a digital spirometer and activity tracker). Furthermore, user satisfaction with the telemedical platform was assessed among patients and health care professionals comprised of the study team (n=5) at the end of the study. We, therefore, exclusively analyzed data from the 30 patients from the intervention group. Secondary objectives of the study (but not part of this analysis) included comparing standard of care with and without additional telemedical intervention. This comparison focused on evaluating clinical endpoints between the control and intervention group. Comprehensive baseline and end-of-study visits were conducted at the study center for both groups and included an in-person clinical assessment of medical history, medication, hospitalizations for MG, care-related questions (eg, accessibility of a specialist or areas of improvement regarding MG treatment), various PROMs and MG-specific clinical assessment through the quantitative myasthenia gravis score (QMGS) [20]. At baseline, the intervention group received a wearable (activity tracker) and a digital spirometer, along with the MyaLink app, which were set up and connected on the patients’ smartphone. Instructions on app and external devices usage were provided by the study team. This enabled remote health monitoring throughout the study by active (PROMs and spirometry) and passive (wearable) continuous data collection. Patients were instructed to fill out PROMs and perform spirometry at predefined intervals. They were instructed to wear the wearable during waking hours (at least 14 h per day) throughout the study period and optionally at night. Telemedical check-ups occurred in weeks 4 and 8, comprising the reviewing of remote monitoring data by study physicians and contacting the patients if necessary (eg, changing medication). Patients could also initiate communication through the chat module at any time, with physicians monitoring messages on weekdays during working hours and responding within 24 hours. Patients completed usability questionnaires through the app a week before the end of the study, while health care professionals conducted their questionnaires after study completion. Furthermore, oral feedback from patients was collected and documented during the end-of-study visit. All data from the study visits and all data from the telemedical check-up was documented in REDCap (Research Electronic Data Capture) software (version 13.7.31; Vanderbilt University).

### Patient-Reported Outcome Measures and Data From App

**Figure 2.**
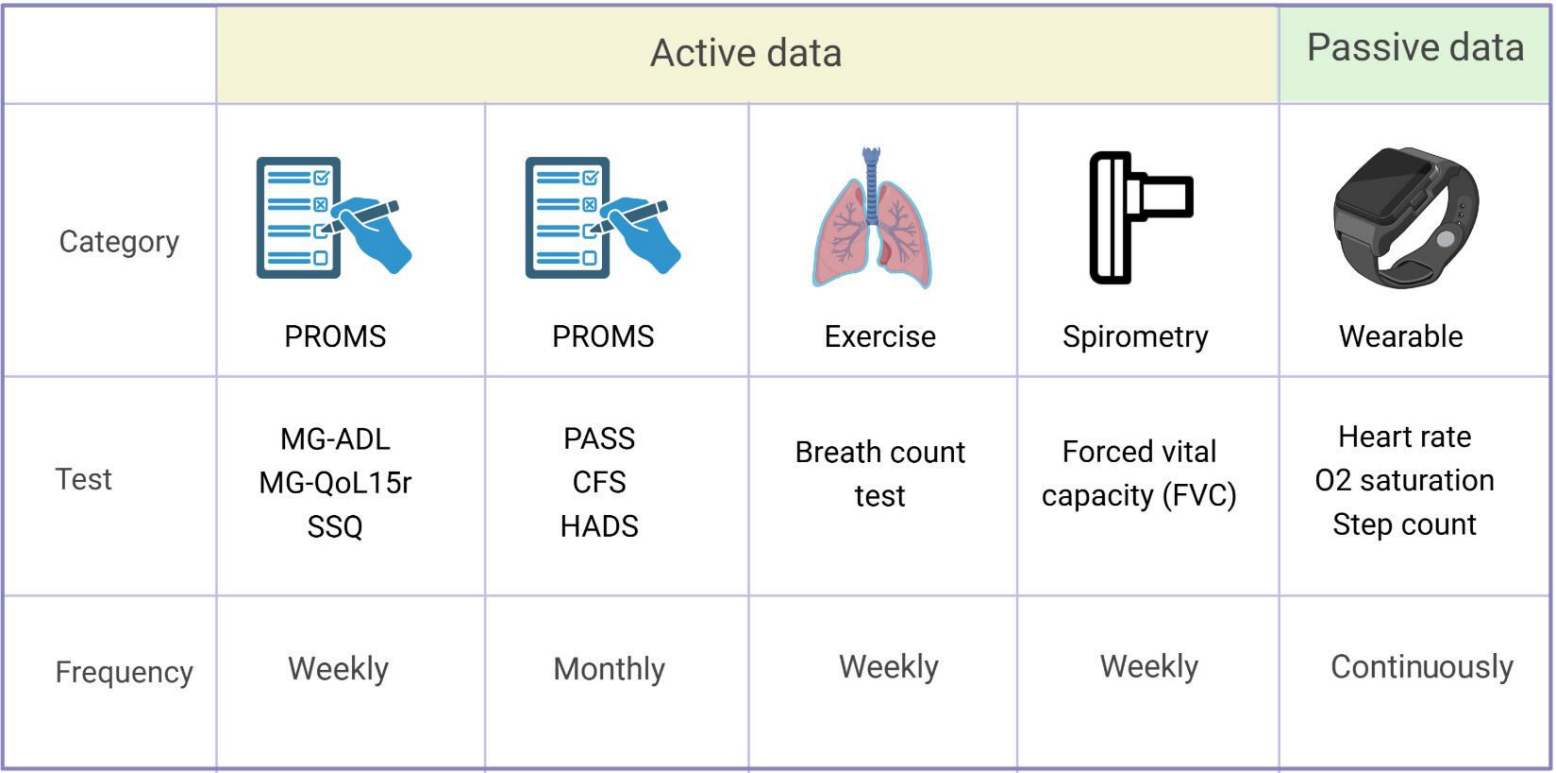
Monitoring plan and frequencies of assessed parameters: Active (PROMs, respiratory function assessment) and passive data (wearable data) were collected during the study period. PROMs data were collected weekly or monthly, depending on the questionnaire. Respiratory function assessment, assessed through breath count test and spirometry, was conducted weekly by the protocol. The collection of wearable data was continuous, with steps aggregated by the hour. PROMs: patient-reported outcome measures; MG-ADL: myasthenia gravis activities of daily living; MG-QoL15r: myasthenia gravis quality of life, revised; SSQ: single simple question; PASS: Patient Acceptable Symptom State; CFS: Chalder Fatigue Scale; HADS: Hospital Anxiety and Depression Scale.

In total, 6 questionnaires and 1 test were assigned to patients through the app, administered weekly or monthly, depending on their specific type (Figure 2). Weekly questionnaires included the myasthenia gravis activities of daily living (MG-ADL) profile [21], myasthenia gravis quality of life, revised (MG-QoL15r) [22], and single simple question (SSQ) [23], which are commonly used in MG studies including large approval studies to assess functional status and quality of life. Patients were also instructed to perform a weekly breath count test [24]. Monthly questionnaires included Chalder Fatigue Scale (CFS 11) [25] and Hospital Anxiety and Depression Scale (HADS) [26] to assess common comorbidities in MG. In addition, Patient Acceptable Symptom State (PASS) was assessed monthly [27]. Furthermore, patients completed 2 user satisfaction questionnaires at the end of the study. For broad comparability, patients completed the System Usability Scale (SUS) [28], where patients ranked each of the 10 statements from 1 to 5 based on how much they agreed with the statement. The second questionnaire was adapted from Braun et al [29] and specifically tailored to the MyaLink telemedical platform. It features 17 items categorized in 3 sections and uses a 4-point Likert scale (strongly agree to strongly disagree). Originally formulated in German for publication purposes, it underwent consensus translation into English by 4 medical health care professionals, including 1 native speaker. After the study, the study team completed a modified version of the questionnaire adapted from Braun et al for health care professionals, respectively.

### Data From External Device or Wearable

Patients were instructed to perform spirometry measurements weekly, with reminders sent through the app through push notifications. The wearable continuously collected oxygen saturation level (every minute), heart rate (every minute), and steps (hourly aggregated). Data from both the digital spirometer and the wearable were transmitted directly to the app by bluetooth. The study team regularly checked the web-based system to ensure receipt of data from external devices and questionnaires, sending reminder messages through the chat module when necessary.

### Analysis

The statistical calculations were performed using R software (version 4.2.2; R Foundation for Statistical Computing) [30]. Descriptive statistics (median [IQR] and absolute and relative frequencies) are presented depending on the scale of the variables.

Feasibility was assessed by adherence rates. For PROMs, assignments of the questionnaire started a week or month after the baseline visit respectively, depending on the questionnaire. Questionnaires were assigned together with a push notification on the patient’s phone and disappeared from the app upon completion or were “overwritten” upon receiving a new assignment. Questionnaires could only be submitted by patients through the app if they were fully completed. Each questionnaire was required to be completed within 7 days to be counted as “completed,” including the monthly assigned questionnaires, with missing entries calculated accordingly. The final day for data acquisition from the last assigned weekly questionnaires ended the day before the end-of-study visit. Total adherence rates were calculated as the total number of completed questionnaires divided by the total number of assigned questionnaires. Additional questionnaire assignments were allowed in cases of clinical deterioration but were excluded from adherence calculations. The study was considered successful if at least 60% of assigned PROMs through the app were answered by patients of the intervention group. Across the 30 patients of the intervention group, a mean adherence rate and a 2-sided 95% CI are determined. If the observed average adherence is at least 76%, with data from 30 patients, the 95% CI would range from 60.7% to 91.3%, thus exceeding the 60% threshold.

Spirometry adherence rates were calculated accordingly (ratio of the total number of actual measurements performed during the study vs total number of measurements required per the study protocol).

Adherence calculations for wearable data included measurements starting the day after the baseline visit and ending the day before the end-of-study visit. Adherence to wearable data from the activity tracker was evaluated by aggregating data over hourly windows, with 100% adherence defined as wearing the device for 14 hours per day per patient (based on the estimated waking hours). This was then extrapolated over the study duration. Each hour necessitated at least one heart rate measurement to validate wear time, considering heart rate a more reliable marker than oxygen saturation. Overall adherence was determined by the sum of the hours the patients had with at least one heart rate measurement divided by sum of the hours per day corresponding to 100% individual adherence.

The user satisfaction questionnaire was descriptively evaluated using the Likert scale. The SUS ranked each of the 10 statements from 1 to 5 based on user agreement, from which a SUS score was calculated: Each patient responses undergo conversion into a new numerical value (0‐4), which is then aggregated and multiplied by 2.5 to transform the original scoring range of 0‐40 into a 0‐100 scale. These scores do not represent percentages but a percentile ranking [31]. Research suggests that a SUS score above 68 is considered acceptable, with scores below 68 classified as not acceptable. Therefore, a score above 68 on the SUS is interpreted as a positive result further supporting the platform’s usability. A more detailed classification considers SUS scores between 73 and 84 as good, between 85 and 99 as excellent, and 100 as the best.

This analysis focuses on the primary study endpoints assessing feasibility and usability. Further exploratory analyses of secondary endpoints, eg, comparative clinical endpoint analyses (between control group and intervention group), evaluations of communication patterns from the MyaLink chat and telemedical interventions are currently prepared and results will be presented in further studies.

### Ethical Considerations

This study received ethics approval by the ethics committee at Charité Universitätsmedizin Berlin (EA2/157/22). Written informed consent was obtained from the study participants before the start of the study. The study was conducted in accordance with the Declaration of Helsinki (2013) and its later amendments. Data storage and handling responsibilities were approved by the ethics committee of Charité Universitätsmedizin Berlin. The data collected through MyaLink is stored on certified data servers, which adhere to a management system according to ISO/IEC 27001:2013.

## Results

### Patient Characteristics

Patients were between 23 and 82 years (median 47.5 years; IQR 40.5-57.5) at time of enrollment. The majority of patients were female (21/30, 70%). Most patients in the study had a mild to moderate disease severity, with 36.7% (11/30) categorized in MGFA II (a or b) status and 43.3% (13/30) in MGFA III (a or b) status at the time of enrolment. Median scores for MG-ADL and QMG were 10 (IQR 5.25-11) and 15.5 points (IQR 12.25-20.75), respectively. On average, patients were diagnosed 5.5 years (IQR 2-9.75) before entry in the study. One patient withdrew from the study due to health reasons unrelated to MG. All results presented include data from the dropout, including measurements taken before the time of dropout (with the patient’s consent). Detailed patient characteristics are presented in Table 1.

**Table 1.** Patient characteristics: age, clinical class, time since diagnosis, study duration, and operating system for intervention group as well as subgroups (female and male).

Characteristics	Overall (n=30)	Female (n=21)	Male (n=9)
Age, median (IQR)	47.5 (40.5‐57.5)	47 (42‐53)	56 (40‐60)
**MGFA^a^ score, n (%)**			
I	1 (3.3)	0 (0)	1 (11.1)
IIa	5 (16.7)	4 (19)	1 (11.1)
IIb	6 (20)	3 (14.3)	3 (33.3)
IIIa	3 (10)	2 (9.5)	1 (11.1)
IIIb	10 (33.3)	8 (38.1)	2 (22.2)
IVa	0 (0)	0 (0)	0 (0)
IVb	5 (16.7)	4 (19)	1 (11.1)
MG-ADL^b^ score, median (IQR)	10 (5.25‐11)	10 (5‐11)	9 (6-12)
MG-QoL15r^c^ score, median (IQR)	15.5 (12.25‐20.75)	17 (12‐21)	13 (13‐19)
QMG^d^ score, median (IQR)	15 (12.25‐17)	15 (13‐18)	14 (10‐15)
Years since MG^e^ diagnosis, median (IQR)	5.5 (2‐9.75)	5 (2-10)	7 (4-9)
**System, n (%)**			
Android	19 (63.3)	15 (71.4)	4 (44.4)
iOS	11 (36.7)	6 (28.6)	5 (55.6)
Days between baseline and end-of-study visit, median (IQR)	84 (84‐85)	84 (83‐85)	84 (84‐86)
Dropout, n (%)	1 (3.3)	1 (4.8)	0 (0)

a MGFA: Myasthenia Gravis Foundation of America.

b MG-ADL: myasthenia gravis activities of daily living.

c MG-QoL15r: myasthenia gravis quality of life, revised.

d QMG: quantitative myasthenia gravis.

e MG: myasthenia gravis.

### Adherence

The total adherence rates for PROMs and external devices data ranged from 74.3%- 97.9% (1830/2464 - 327/334) across all data types and are detailed in Table 2. When examining adherence by data category, adherence to PROMs was the highest, followed by spirometry, and then wearable data from the activity tracker.

**Table 2.** Total adherence rates for different data groups.

Data group	Adherence (total completed/total assigned), n/N (%)
**Overall PROMs^a^**	1139/1179 (96.6)
MG-ADL^b^	321/334 (96.1)
MG-QoL15r^c^	325/334 (97.3)
SSQ^d^	327/334 (97.9)
CFS^e^	55/59 (93.2)
HADS^f^	5/59 (93.2)
PASS^g^	56/59 (94.9)
Breath count test	326/334 (97.6)
**Spirometry**	
FVC^h^	293/334 (87.7)
**Wearables (at least 14 h per day)**	
Pulse	1830/2464 (74.3)
Pulse (excluding days without any wearable measurement from the analysis)	1830/2261 (80.9)

a PROMS: patient-reported outcome measures.

b MG-ADL: myasthenia gravis activities of daily living.

c MG-QoL15r: myasthenia gravis quality of life, revised.

d SSQ: single simple question.

e CFS: Chalder Fatigue Scale.

f HADS: Hospital Anxiety and Depression Scale.

g PASS: Patient Acceptable Symptom State.

h FVC: forced vital capacity.

**Figure 3.**
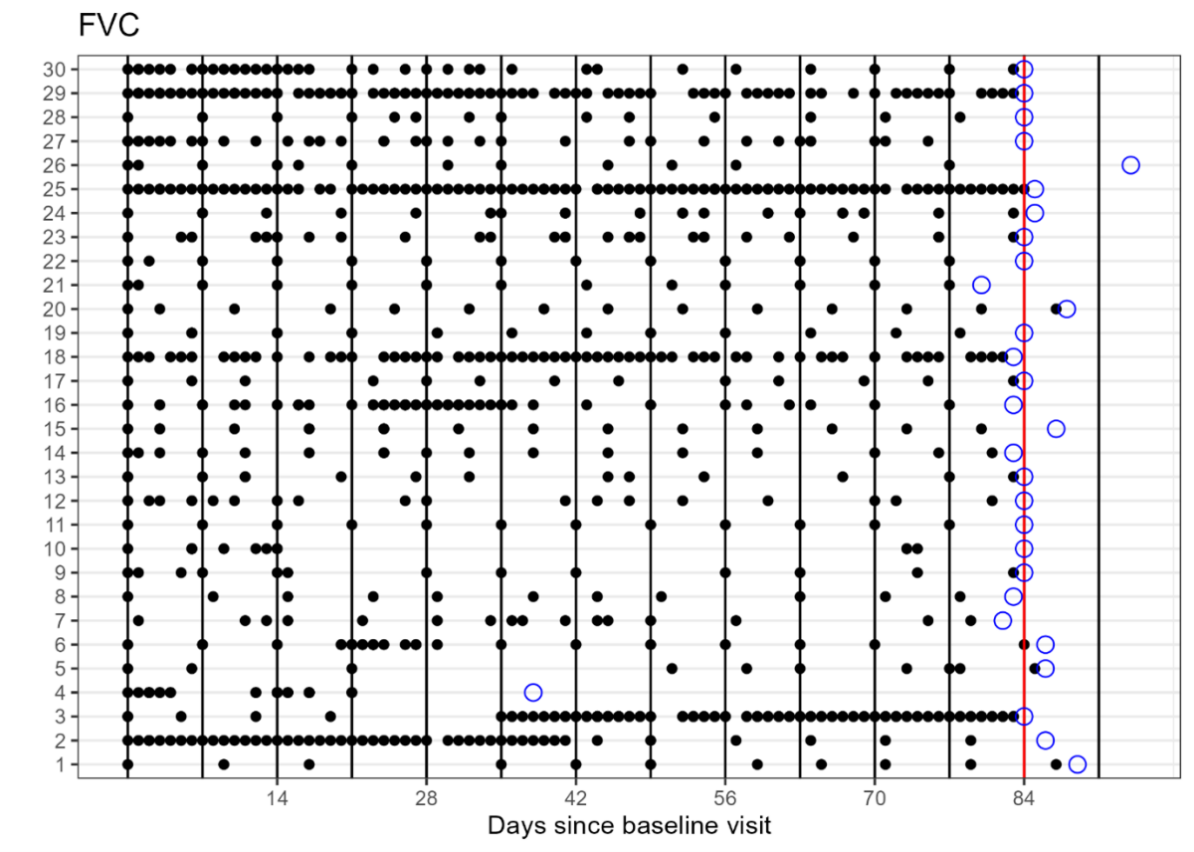
Adherence of spirometry measurements: The blue circles represent the end-of-study visit for each patient, while the red line indicates week 12, marking the duration of the study. Each black vertical line indicates when a spirometry measurement was assigned according to the study protocol (weekly intervals). Patients were given a 7-day window until the next weekly assignment for measurement to qualify as “completed.” The black dots mark the times when the patient actually performed the spirometry measurement. Each measurement from the digital spirometer was timestamped and subsequently automatically transmitted to the app through bluetooth, with the values being integrated accordingly. FVC: forced vital capacity.

Adherence to PROMs ranged between 93.2% (55/59) and 97.9% (327/334), depending on the specific questionnaire. Patients were assigned weekly questionnaires and spirometry measurements between 11 and 13 times (drop out: 5 times) during the study, depending on individual study duration of a participant. Monthly questionnaires were assigned twice to all patients (drop out: 1). There was no difference in adherence regarding the questionnaire frequency. For the breath count test, adherence was 97.6% (326/334). Adherence to spirometry was slightly lower than PROMs (293/334, 87.7%), likely due to the higher task burden associated with these active measurements (Figure 3). Patients conducted a median of 49 spirometry measurements (IQR 15-59).

**Figure 4.**
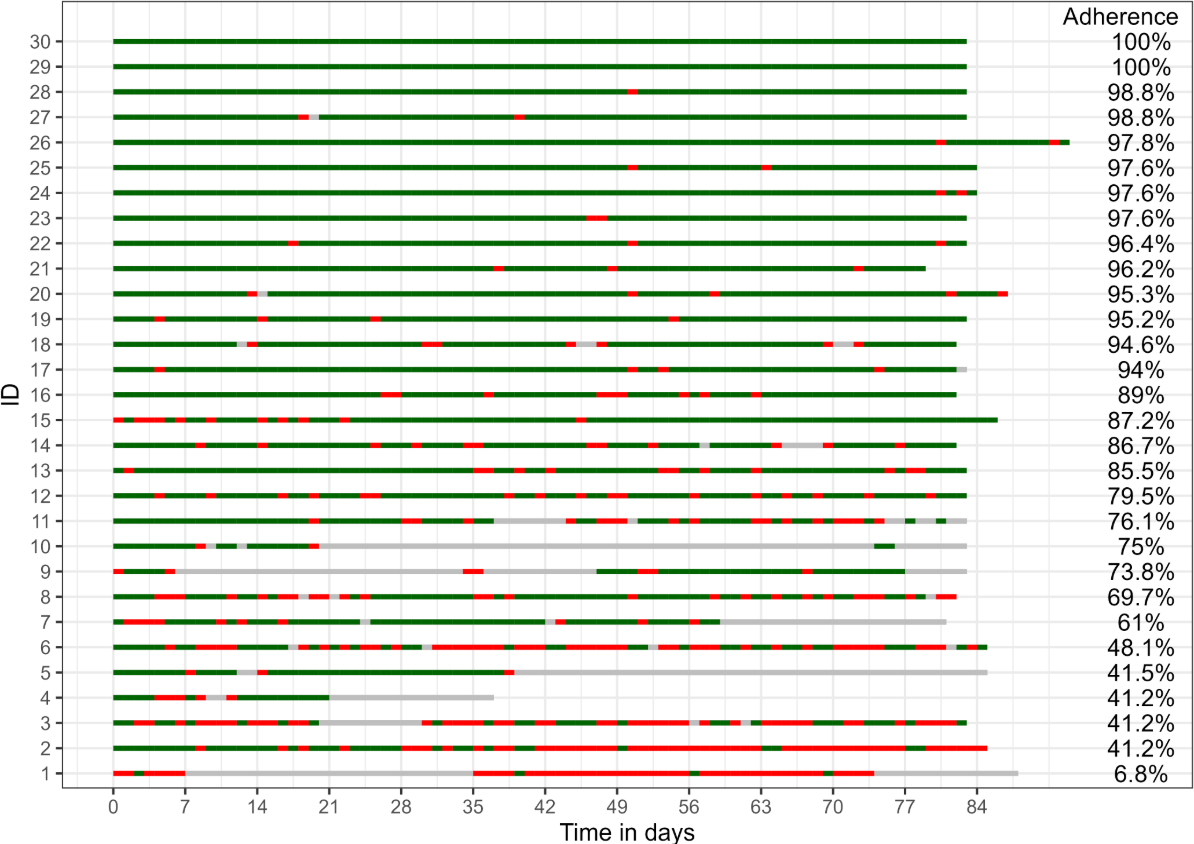
Adherence rates for wearable data from the activity tracker: Days since baseline visit on the X-axis, with patients represented on the Y-axis and the adherence rate per patient in the column on the right side. Days with wearable usage of at least 14 hours per day were classified as compliant (marked in green). A wearable hour was counted if at least one heart rate measurement value was recorded per hour. Days with less than 14 hours of wearable usage per day were marked as red. Days without any recorded heart rate measurements were marked in gray and excluded from the analysis.

On average, patients wore the wearable for a median duration of 20 hours per day (IQR 15‐24). Wearable adherence rates exhibited considerable variability among patients, ranging from 6.8% (4/59) to 100% (83/83) (Figure 4), despite an overall strong adherence of 80.9% (1830/2261) for all patients during the study when excluding days without any data input (no recorded heart rate measurements) from the calculation (Table 2). Including days without wearable data input to the calculation decreased adherence to 74.3% (1830/2464). Clinical insights from chat interactions, as evidenced by the timestamps within the chat messages, unveiled several factors impacting patients with decreased device use. For example, out of the 30 patients, 2 patients (6.7%) experienced a myasthenic crisis necessitating prolonged hospitalization and reported having forgotten the devices at home. Furthermore, 1 patient (3.3%) had difficulty with using the app (older patient who was overwhelmed by the technical operation and conducting measurements with the digital spirometer) with numerous interactions with the software provider’s support team. Some patients experienced discomfort with the rubber strap of the activity tracker. Some patients had device-related issues such as (recurring) pairing problems and 1 out of the 30 patients (3.3%) received a defective activity tracker, necessitating a device replacement. However, even after replacement the patient reported ongoing connectivity issues between the wearable and his smartphone.

### User Satisfaction

**Figure 5.**
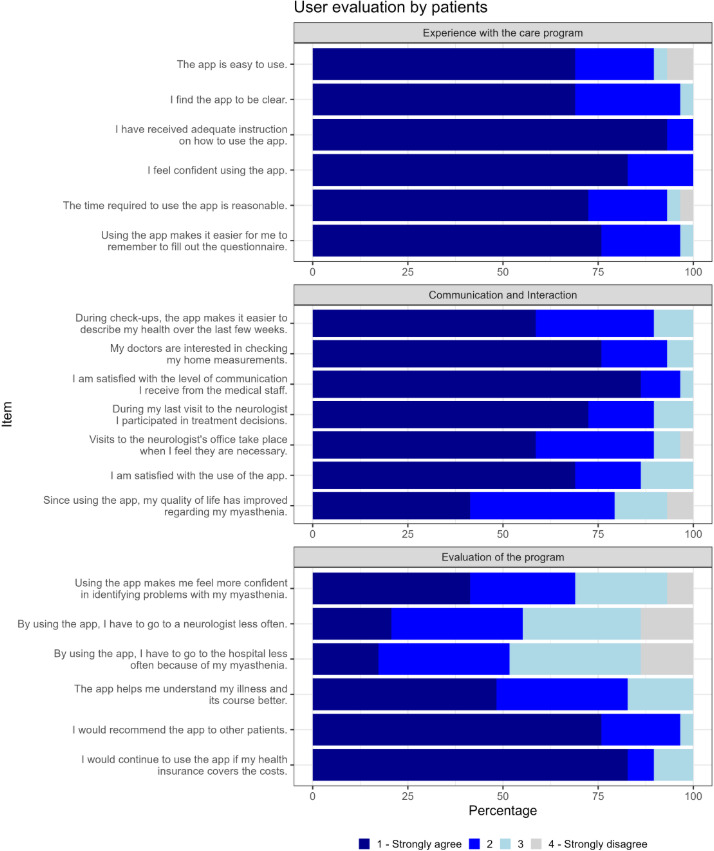
User evaluation for patients: The questionnaire was divided into 3 categories: “Experience with the care program,” “Communication and Interaction” and “Evaluation of the program.” All 17 questions could be answered using a 4-point Likert scale ranging from 1 (strongly agree) to 4 (strongly disagree).

**Figure 6.**
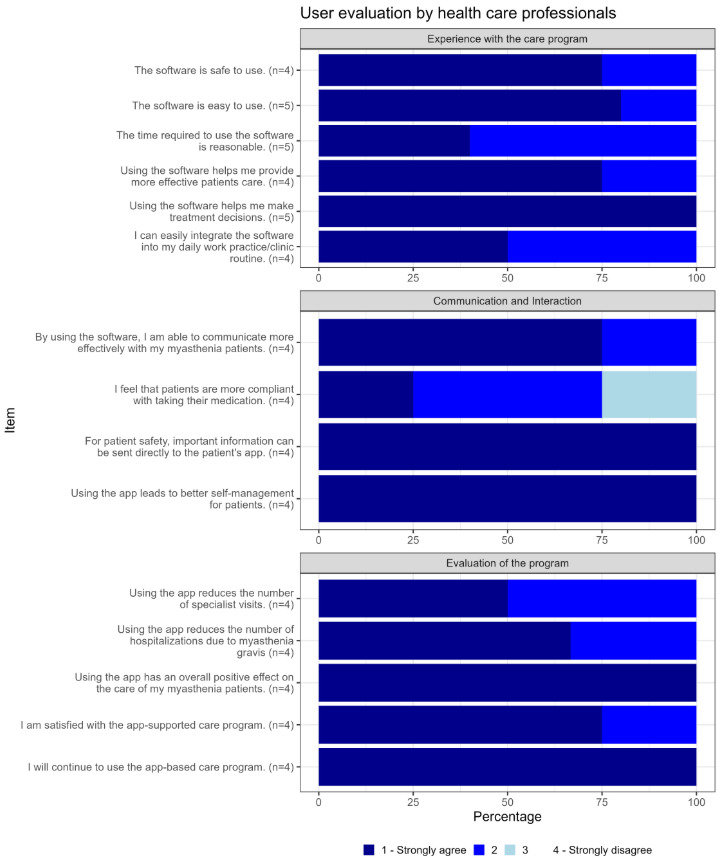
User evaluation for health care professionals: The questionnaire was divided into 3 categories: “Experience with the care program,“ “Communication and Interaction,” and “Evaluation of the program.” All 17 questions could be answered using a 4-point Likert scale ranging from 1 (strongly agree) to 4 (strongly disagree). Depending on the role within the study team (eg, physician and study nurse), only certain questions in the questionnaire could be answered.

The highest levels of agreement were observed in the following order: experience with the care program, communication, and evaluation of the program. Regarding the care program evaluation, 89.7% (26/29) agreed that the app was easy to use while 93.1% (27/29) agreed that the time investment required for app usage was appropriate. In total, 89.6% (26/29) of patients agreed that describing their health status during follow-up appointments was easier. A total of 93.1% (27/29) indicated that treating physicians were interested in reviewing the collected monitoring parameters. In addition, 79.3% (23/29) of patients agreed that their quality of life had significantly improved since app usage. Futhermore, 96.6% (28/29) expressed willingness to recommend the app to other patients. In addition, 69% (20/29) agreed that they felt more confident in identifying MG-specific problems using the app. Views on the reduction of visits to neurologists or clinics were mixed; 55.2% (16/29) agreed to needing fewer visits to neurologists, while 51.7% (15/29) agreed to reduced visits to clinics, with 13.8% (4/29) strongly disagreeing with both categories (Figure 5). Out of the 29 patients, 14 patients (46.7%) responded that they did not agree that they required less frequent hospital visits; of those 14 patients, 8 patients (57.1%) were hospitalized during the study period due to MG (two of whom were in intensive care). In addition, 7 of these 14 patients (50%) experienced at least one exacerbation. Results of user evaluation from the health care professionals showed similar high acceptance rates regarding the telemedical platform, ease of use, and overall positive evaluation on the effect on patient care (Figure 6).

**Figure 7.**
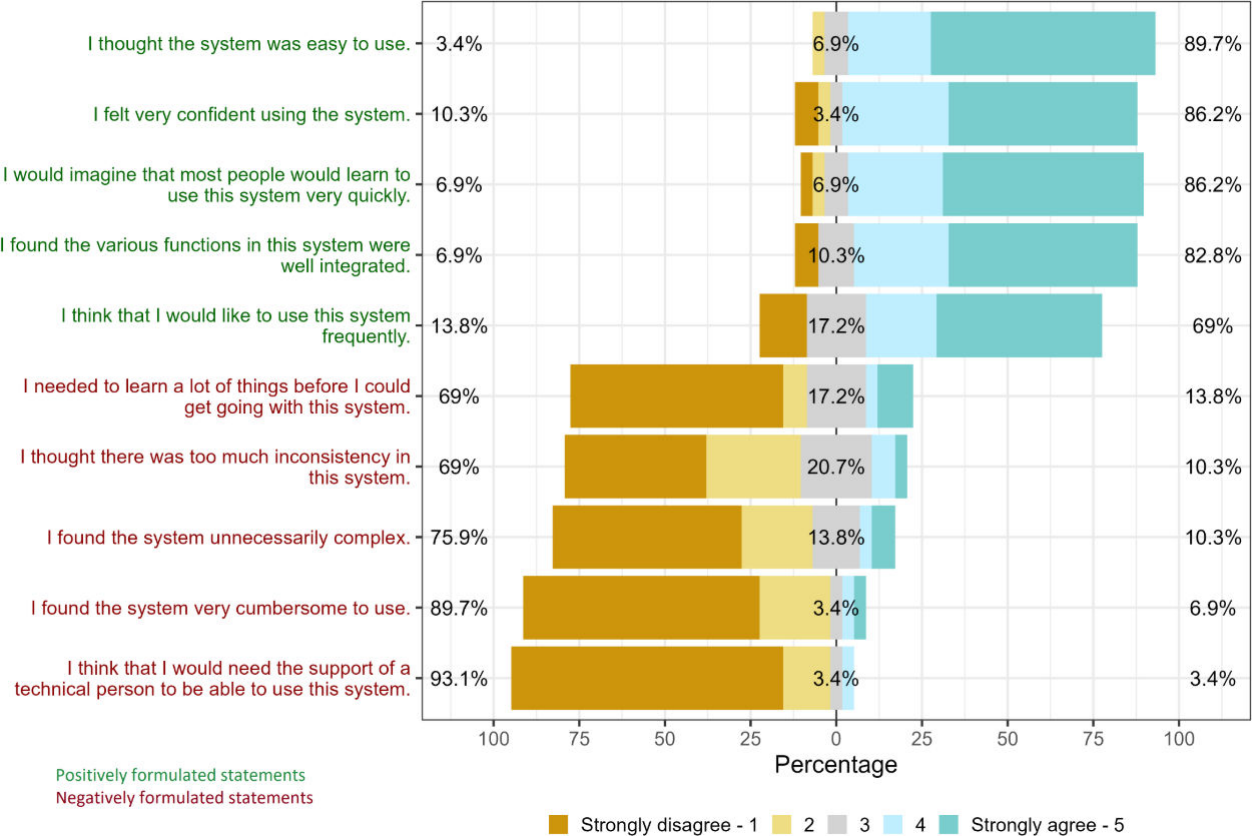
The System Usability Scale (SUS) uses a 10-item questionnaire with 5 response options for respondents, ranging from strongly agree to strongly disagree. Due to rounding, the percentage for Question 2 (“I feel confident using the app”) is 99% instead of 100%.

The overall median SUS score was 85 (IQR 75-90). Figure 7 shows that there was a high level of agreement regarding the system’s ease of use (26/29, 90%), patients’ confidence in operating it (25/29, 86%), and patients’ belief that others would quickly learn to use the app (25/29, 86%). Notably, 21% (6/29) remained neutral regarding perceived inconsistencies in the app, although the majority (20/29, 69%) tended to disagree. Among patients (N=22) who responded at least once with a neutral (Likert-scale 3) or negative response to any question (agreeing or strongly agreeing with negatively formulated questions or disagreeing or strongly disagreeing with positively formulated questions), there were 14 females (63.6%) with a median age of 47.5 (IQR 39.25-61.25; mean 49.7, SD 15.70), and the MG-ADL score at baseline was median 9.5 (IQR 5-11; mean 8.5, SD 3.60). Of all 29 patients, 69% (20/29) expressed willingness to use the app regularly. Among the patients who responded negatively or neutral to this statement, the documented oral feedback collected at the end-of-study visit mainly encompassed technical feedback, such as suggestions for adjusting the size of the chat window, implementing filter options for monitoring parameters in the health diary, and presenting preferences for the parameter dashboard. Furthermore, patients also frequently noted necessary improvements for a more user-friendly medication plan. In addition, there were critiques regarding the omission of specific symptoms in the PROMs (eg, nonmotor symptoms, pain, and sleep) and requests from patients for a free-form diary function (eg, for documenting infections or daily fluctuations).

## Discussion

Our study evaluated the feasibility and usability of a telemedicine platform for patient-physician interaction and monitoring for MG, incorporating wearable data collection. Feasibility, measured by adherence, ranged from 74.3%- 97.9% (1830/2464 - 327/334) across all data types, with the highest adherence observed for completion of PROMs (1139/1179, 96.6%) within the app, followed by adherence to spirometry measurements (293/334, 87.7%), and then wearable adherence (1830/2261, 80.9%). Next to adherence, user satisfaction among patients and health care professionals was assessed and generally rated high to very high across different categories (care program, communication, and evaluation of the program) and considered “excellent” according to the SUS score. The majority of patients stated their willingness to continue using the app.

Our results demonstrate that the introduction of such a digital tool in a clinical setting is feasible and facilitates clinical monitoring regardless of age or gender. These findings represent the first results in the field of MG for telemedical intervention. There was mutual acceptance from both health care professionals and patients, as evidenced by the high adherence rates and good to very good user satisfaction assessed at the end of the study.

Overall, patients demonstrated greater engagement with the app questionnaires compared with data collection through wearables and spirometry. Passive data collection by wearables displayed higher adherence rates than active spirometry, interestingly diverging from previous research [32]. Consistent with previous clinical studies [33-35], there was a variability particularly in wearable adherence. Our additional clinical data from the chat interactions revealed that commonly technical issues (eg, pairing problems and device malfunction) and discomfort (eg, wristband intolerance) were factors contributing to lower adherence rates for some patients. Only 1 patient consistently experienced difficulties (and ultimately discontinued wearable use). However, patients wore the device significantly longer than required by the study protocol, including during nighttime (median 20 h per day (IQR 15-24)). Similarly, spirometry was performed more frequently than required by protocol (49 measurements (median; IQR 15-59)). This indicates a high need and willingness of patients to monitor their own symptoms. Our study results align with broader research on telemedicine for chronic diseases, demonstrating high feasibility and user acceptance, consistent with findings in other disorders. Similar to studies in palliative care in cancer patients [36], depression monitoring [37] and heart failure [38], our results confirm the feasibility of continuous monitoring through mobile technologies for chronic conditions. With an adherence rate of 80.9% (1830/2261) for wearables, our study falls at the higher end of reported ranges (53%‐94%) [36-43]. However, the higher adherence rates for active data (PROMs) over passive data (wearables) in our results contrasts with other studies finding [40,42], indicating potential disease-specific differences requiring further investigation. In addition, patients’ sense of control during active data input or technical challenges with wearables could have affected adherence to passive monitoring. Still, comparison with other studies for different diseases remains difficult, as literature on wearable adherence expose inconsistency in reporting and study design.

Overall user satisfaction was high, particularly patients agreed on the ease of use, satisfaction, and improvement in understanding their symptoms. This finding aligns with a study in multiple sclerosis that reported sustained satisfaction with remote monitoring using smartphone and wearable technology over 6 months [40]. Further qualitative analyses, such as focus groups with patients, could aid in gaining a deeper understanding of the reasons behind these perceptions and to identify areas for improvements. First approaches have been made by the research team within the framework of additional small-scale pilot phases involving patients testing the platform use and workshops for feedback collection and development requests. Furthermore, mixed opinions were indicated by patients regarding whether the use of the telemedicine platform could reduce visits to neurologists or clinics, indicating that the platform may primarily serve as a complement to existing care structures rather than a replacement of personal interactions.

For successful implementation in clinical care, the efficient usage and integration of such data into clinical decision-making processes will be essential. This includes logistical efforts, but also necessitates a careful assessment of data quality and interpretation, and the “appropriate” monitoring intensity for different patient groups (eg, those with a short disease duration, highly active disease, and fast-acting treatment regimens may require more frequent assessments). Furthermore, addressing regulatory challenges, particularly regarding reimbursement structures for health care professionals will be crucial for long-term integration within health care systems.

### Limitations

Our study encountered several challenges associated with remote monitoring technology: These included missing passive wearable data primarily due to technical issues and synchronization problems and patients’ discomfort with the wristband from the wearable. Addressing these challenges and providing solutions, such as training sessions and available technical support, is crucial to ensure optimal patient experience and adherence in future studies using these technologies. Furthermore, the study cohort is relatively small, and the study duration is short. Patients with higher digital literacy may have been more likely to participate, potentially impacting the representativeness of the cohort. Regarding user evaluation of health care professionals, it should be noted that the study team size was small and partially comprised members of the development team for the platform, which could add to bias in answering the questionnaire.

### Conclusion

In conclusion, our results demonstrate the feasibility of remote monitoring for collecting both subjective (active) and objective (passive) data from multiple sources among patients with MG, as evidenced by above-average adherence rates and acceptability. This underscores the potential for user-friendly digital tools for long-term monitoring of patients with MG and their willingness to track disease progression. Larger and prolonged studies are needed to benchmark our findings and to further explore the effective implementation of such tools and their clinical implications in practice.

## Abbreviations

BlnDSG: Berlin Data Protection Act
CFS: Chalder Fatigue Scale
GPDR: General Data Protection Regulation
HADS: Hospital Anxiety and Depression Scale
MG: myasthenia gravis
MG-ADL: myasthenia gravis activities of daily living
MG-QoL15r: myasthenia gravis quality of life, revised
MGFA: Myasthenia Gravis Foundation of America
PASS: Patient Acceptable Symptom State
PROMs: patient-reported outcome measures
QMGS: quantitative myasthenia gravis score
RCT: randomized controlled trial
REDCap: Research Electronic Data Capture
SSQ: single simple question
SUS: System Usability Scale
